# Synergistic antibacterial activity of baicalin in combination with oxacillin sodium against methicillin‐resistant *Staphylococcus aureus*

**DOI:** 10.1002/2211-5463.13952

**Published:** 2024-12-15

**Authors:** Xin Meng, Mengna Kang, Zhiyun Yu, Changyou Li, Yang Chen, Taicheng Jin, Kai Wang, Haiyong Guo

**Affiliations:** ^1^ College of Life Science Jilin Normal University Siping China; ^2^ RemeGen Co., Ltd. Yantai China

**Keywords:** baicalin, biofilm, membrane, methicillin‐resistant *Staphylococcus aureus*, oxacillin sodium, synergism

## Abstract

Methicillin‐resistant *Staphylococcus aureus* (MRSA) poses a challenge for clinical treatment and combining antibiotics with other agents might be a promising strategy to overcome this challenge. This study explored the synergistic antibacterial activity of baicalin (traditional Chinese medicine extract) and the narrow‐spectrum beta‐lactam antibiotic oxacillin sodium, both of which are poorly active against MRSA *in vitro*. The combination of baicalin and oxacillin sodium showed a synergistic effect with a fractional inhibitory concentration index of 0.5. Mechanistically, the supplementation of baicalin increased the permeability of bacterial cell walls and cell membranes, enhancing oxacillin sodium entry and bactericidal action. The combination of baicalin and oxacillin sodium also significantly inhibited MRSA USA300 biofilm formation by further reducing polysaccharide intercellular adhesion production. Therefore, the combination of baicalin and oxacillin sodium offers a new therapeutic option for addressing clinical MRSA resistance. Further studies, including clinical trials, will be required to validate the observed *in vitro* results.

## Graphical abstract

The bacterial cell membrane and cell wall are the primary targets of baicalin. Baicalin increases the permeability of bacterial membranes, allowing oxacillin sodium to easily penetrate the cells and effectively exert its antimicrobial activity.

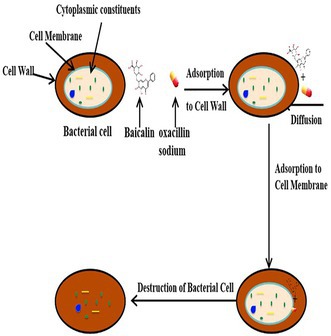

**Abbreviations**
AKP: Alkaline phosphatase
CLSM: confocal laser scanning microscopy
CR: Congo red
CV: crystal violet
EDTA: ethylene diamine tetraacetic acid
FICI: fractional inhibitory concentration index
HEPES: *N*‐2‐hydroxyethylpiperazine‐*N*‐ethane‐sulphonic acid
MICs: minimum inhibitory concentrations
MRSA: methicillin‐resistant *Staphylococcus aureus*
NCCLS: National Committee for Clinical Laboratory Standards
ONPG: 2‐nitrophenyl‐β‐d‐galactopyranoside
PBP: penicillin‐binding protein
PBS: phosphate‐buffered saline
PI: propidium iodide
PIA: polysaccharide intercellular adhesion
SCCmec: staphylococcal cassette chromosome mec
SD: standard deviation
SEM: scanning electron microscopy
TCM: traditional Chinese medicine
TSA: tryptic soya agar
TSB: tryptic soy broth

In recent years, the widespread use of antibiotics has led to a significant increase in antibiotic‐resistant bacteria, posing challenges for healthcare systems worldwide [1]. The emergence of methicillin‐resistant *Staphylococcus aureus* (MRSA), which causes serious and even fatal infections, is a major threat to clinical treatment [2, 3]. In this context, people pay more attention to common antibiotics such as oxacillin sodium. Oxacillin sodium is a β‐lactam drug that inhibits the synthesis of bacterial cell walls by binding to penicillin‐binding protein (PBP). Unfortunately, the *mecA* gene of *Staphylococcus aureus* encodes an additional penicillin‐binding protein, PBP2a, that reduces affinity for oxacillin sodium [4]. The *mecA* gene is located on a mobile genetic element called the staphylococcal cassette chromosome *mec* (SCC*mec*), which can transfer between different strains of *Staphylococcus aureus*, facilitating the spread of MRSA [5]. Therefore, there is an urgent need to accelerate the development of new antimicrobial drugs as alternatives to antibiotics [6]. However, the discovery and development of new drugs are time‐consuming and have low success rates [7]. Additionally, long‐term use of newly developed drugs targeting a single mechanism in a clinical setting can lead to the emergence of new resistant strains [8]. To enhance the therapeutic potential of existing antibiotics, combining them with other biological or chemical molecules is a promising approach. This combination strategy not only significantly enhances antibacterial activity but also effectively reduces the development of bacterial resistance [9, 10]. Additionally, it further allows for a reduction in the required dosage of antibiotics and minimizes adverse effects [11]. Consequently, it offers an effective approach to overcoming antibiotic toxicity and resistance, providing robust support for safer and more effective antimicrobial treatments.

Many traditional Chinese medicine (TCM) extracts exhibit antimicrobial properties and have gained attention in the medical field due to their low adverse effects and potential to reverse bacterial resistance [12, 13]. While many TCM extracts exert their antimicrobial activity by targeting bacterial membranes, their slow effects and long treatment periods have hindered their development as clinical drugs [14, 15, 16]. Previous studies have shown that combining certain TCM extracts with conventional antibiotics results in greater antibacterial activity than either agent alone and helps prevent microbial resistance [17, 18]. Therefore, the synergistic effects between TCM extracts and antibiotics can improve the effectiveness of antibiotics, offering a promising new approach to address bacterial resistance. Baicalin is a flavonoid compound primarily derived from the root of the traditional Chinese medicinal *Scutellaria baicalensis Georgi*. It exhibits a range of biological activities, including antitumor, antibacterial, anti‐inflammatory, neuroprotective, antidiabetic, antiepileptic, and antioxidant [19, 20, 21]. Currently, there are limited reports on its antibacterial mechanism as an antibiotic adjuvant [22, 23, 24]. The aim of this study was to determine whether baicalin has a synergistic effect with antibiotics, thereby creating a more effective method for treating resistant bacteria. We used the checkerboard method to investigate the enhanced effects of baicalin and oxacillin sodium combined treatment on the USA300 strain. Additionally, we explored the potential mechanisms of this synergy *in vitro*.

## Materials and methods

### Bacterial strains, cell cultures, and chemicals

The community‐acquired methicillin‐resistant *Staphylococcus aureus* (CA‐MRSA) USA300 strains were provided by Professor Ji from the University of Minnesota, USA. All USA300 isolates were incubated on tryptic soy agar (TSA, HopeBio, Qingdao, China) and cultured aerobically for 24 h at 37 °C. A bacterial colony was inoculated into 5 mL of tryptic soy broth (TSB, HopeBio) and cultured to the logarithmic phase (1 × 10^8^ CFU·mL^−1^) for 8 h at 37 °C. Sterile water served as the negative control, while gentamicin was used as the positive control in our experiments. Oxacillin sodium, gentamicin, and 2‐Nitrophenyl‐β‐d‐galactopyranoside (ONPG) were purchased from Aladdin, Shanghai, China. Baicalin (HPLC ≥ 98%), Congo red (CR), and crystal violet (CV) were obtained from Solarbio, Beijing, China. The BCA Protein Assay Kit was purchased from Bestbio (Nanjing, China). The Alkaline Phosphatase (AKP) Assay Kit and LIVE/DEAD BacLight™ Bacterial Viability Kit were procured from Nanjing Jiancheng Bioengineering Institute (Nanjing, China) and Invitrogen (Life Technologies, Shanghai, China), respectively.

### Determination of MICs

The minimum inhibitory concentrations (MICs) of oxacillin sodium and baicalin against the USA300 strain were determined using a 96‐well microtiter plate following the standard microdilution method [25]. In brief, baicalin was first solubilized with a trace amount of 5% NaHCO_3_ (pH 7.2–7.4) and then serially diluted twofold with sterile water to achieve concentrations ranging from 156 to 5000 μg·mL^−1^. Gentamicin and oxacillin sodium were also serially diluted twofold with sterile water, with the concentration range for oxacillin sodium being 7.8 to 250 μg·mL^−1^ and for gentamicin being 0.78 to 25 μg·mL^−1^. Then, 50 μL of drug was added to the wells of a 96‐well microtiter plate and mixed with 50 μL of bacterial culture in the logarithmic phase (2 × 10^5^ CFU·mL^−1^), followed by overnight incubation at 37 °C. The growth of the cultures was measured at 600 nm using a Spark™ multimode microplate reader (Tecan (Shanghai) Laboratory Equipment Co., Shanghai, China). The MIC was defined as the lowest concentration of the drug that inhibited 95% of bacterial growth [8].

### Checkerboard microbroth assay

The combined effects of baicalin and oxacillin sodium were evaluated using the checkerboard broth dilution method on a 96‐well microplate [26]. In brief, the concentrations of baicalin and oxacillin sodium started from their respective MICs. Baicalin was subjected to a twofold serial dilution along the *x*‐axis on the plate to create six concentration gradients (from 1250 μg·mL^−1^ down to 0 μg·mL^−1^), while oxacillin sodium was subjected to a twofold serial dilution along the *y*‐axis to create six concentration gradients (from 31.3 μg·mL^−1^ down to 0 μg·mL^−1^). The USA300 strain was added to achieve a final concentration of 1 × 10^5^ CFU·mL^−1^, and the plates were incubated at 37 °C for 24 h. The optical density at 600 nm was measured using a Spark™ multimode microplate reader (Tecan (Shanghai) Laboratory Equipment Co.). The interaction between baicalin and oxacillin sodium was determined by calculating the fractional inhibitory concentration index (FICI) using the formula [27]: FICI = FIC (A) + FIC (B), where FIC (A) = MIC of baicalin in combination/MIC of baicalin alone, and FIC (B) = MIC of oxacillin sodium in combination/MIC of oxacillin sodium alone. The FICI values were interpreted as follows: synergistic effect, ≤ 0.5; additive effect, > 0.5 to 1; no interaction, > 1 to < 4; antagonistic effect, ≥ 4. Additionally, the antimicrobial effect of the combination of gentamicin (six concentration gradients were established, ranging from 1.56 μg·mL^−1^ down to 0 μg·mL^−1^) and baicalin was assessed according to the aforementioned methodology.

### Growth curve assay

The USA300 strain in logarithmic phase was diluted in sterile TSB to a concentration of 2 × 10^5^ CFU·mL^−1^. A 100 μL of the bacterial solution was added to each well of a 96‐well microplate containing 100 μL of either the sterile water (untreated control), baicalin (final concentration of 1/4 × MIC), different concentrations of oxacillin sodium (final concentration of 1/4 × MIC, 1 × MIC, 2 × MIC, respectively), or a mixture of baicalin (final concentration of 1/4 × MIC) and different concentrations of oxacillin sodium. The microplate was then placed in a Bioscreen C automatic microbial growth curve analyzer (Shanghai Weizai Business Development Co. Shanghai, China) and monitored at 37 °C every 2 h for a total of 12 times. Gentamicin was used as a positive control. Growth curves were plotted with time as the *x*‐axis and the difference between the monitored absorbance value and the initial absorbance value as the *y*‐axis to analyze the impact of the combination treatment on the growth of USA300 [28].

### Bactericidal kinetics assay

The bactericidal rate of baicalin and oxacillin sodium in combination against USA300 was evaluated using the method described by Rady *et al*. [29]. Oxacillin sodium (final concentration of 1/4 × MIC, 1 × MIC, 2 × MIC) and baicalin (final concentration of 1/4 × MIC) were incubated separately or in combination with 2 × 10^5^ CFU·mL^−1^ bacterial suspension at 37 °C for specified time intervals (0, 30, 60, 90, 120, and 180 min). Sterile water was used as negative control, and gentamicin was used as positive control. The cultures were washed twice with sterile TSB, the surviving bacteria were subjected to serial dilutions by factors of 10, with the control group diluted 10^4^‐fold and the drug treatment group diluted 10^2^‐fold, before being spread on tryptic soy agar plates. Colony counts were performed after 24 h of incubation at 37 °C. Bactericidal kinetics curves were plotted to show the changes in the number of viable bacteria over time following treatment with the combination of baicalin and oxacillin sodium against USA300. The bactericidal kinetics experiment of baicalin in combination with gentamicin was conducted according to the aforementioned method.

### Cell membrane permeability assay

When cell membrane permeability changes, intracellular proteins can leak into the extracellular environment [30, 31]. Therefore, protein leakage experiments are used to assess the impact of the combination of baicalin and oxacillin sodium on the permeability of USA300 cell membranes. The bacterial cultures were centrifuged at 1484 ***g*** ·min^−1^ for 10 min, and the supernatant was discarded. The cells were washed twice with sterile phosphate‐buffered saline (PBS), then resuspended, and diluted to 1 × 10^6^ CFU·mL^−1^ with PBS. The test drugs (1/4 × MIC baicalin alone; 1/4 × MIC, 1 × MIC, 2 × MIC oxacillin sodium alone; 1/4 × MIC baicalin was combined with 1/4 × MIC, 1 × MIC, 2 × MIC oxacillin sodium, respectively) were added, and the mixture was incubated at 4 °C with shaking at 100 r.p.m.·min^−1^ for 6 h. After incubation, the samples were centrifuged again at 1484 ***g*** ·min^−1^ for 10 min, and the supernatant was collected [32]. Sterile water and gentamicin were used as negative and positive controls, respectively. The amount of leaked protein was determined using a BCA Protein Assay Kit (Bestbio, Nanjing, China) following the manufacturer's instructions.

### Determination of alkaline phosphatase (AKP) activity

According to the method of Chen *et al*. [33], logarithmic phase bacteria were diluted to 1 × 10^6^ CFU·mL^−1^ and incubated at 37 °C for 2 h with sterile water, baicalin (final concentration of 1/4 × MIC), different final concentrations of oxacillin sodium (1/4 × MIC, 1 × MIC, 2 × MIC), or a mixture of baicalin (final concentration of 1/4 × MIC) and oxacillin sodium at different final concentrations (1/4 × MIC, 1 × MIC, 2 × MIC). Gentamicin was used as a positive control. The cultures were then centrifuged at 1000 ***g*** for 10 min, and the supernatant was collected. The activity of AKP was measured using an AKP Assay Kit (Jiancheng Bioengineering Institute, Jiangsu, China), following the manufacturer's instructions. One king μnit of AKP activity was defined as 1 mg of phenol produced by 100 mL of culture solution interacting with the matrix in a 15‐min interval [34].

### Preparation of bacterial protoplasts

Log phase bacteria (1 × 10^8^ CFU·mL^−1^) were harvested by centrifugation and washed twice with 10 mm phosphate buffer (pH 7.0). They were then resuspended in phosphate buffer containing 0.5 m sucrose. Lysozyme was added to the cell suspension at a final concentration of 80 μg·mL^−1^, followed by incubation at 37 °C with shaking for 2 h. After centrifugation at 835 ***g***. for 5 min, the cells were resuspended in a phosphate buffer and Ethylene Diamine Tetraacetic Acid (EDTA) mixture (volume ratio 1 : 1) and incubated at 37 °C for 15 min. Successful separation of the protoplasts from the cell wall was indicated by over 95% of cells staining red with Gram stain [35]. Protoplasts were then collected by centrifugation, washed with phosphate buffer containing 0.25 m sucrose, and resuspended in a phosphate buffer containing 5 mm *N*‐2‐hydroxyethylpiperazine‐*N*‐ethane‐sulphonic acid (HEPES), 20 mm glucose, and 100 mm KCl (pH 7.0). Gentamicin was used as a positive control. The determination of MIC and the checkerboard microbroth assays for the protoplasts were conducted exactly as described for the intact bacteria [36].

### Scanning electron microscopy

The effects of the combination of baicalin and oxacillin sodium on the cell morphology of the USA300 strain were observed using scanning electron microscopy (SEM). Log phase bacterial cultures (1 × 10^8^ CFU·mL^−1^) were incubated at 37 °C for 1 h with either baicalin (1/4 × MIC), oxacillin sodium (1/4 × MIC and 1 × MIC), or a combination of both. Sterile water as a negative control. Bacterial cells were collected by centrifugation at 835 ***g*** for 10 min, washed three times with phosphate buffer, and then fixed overnight at 4 °C in 2.5% glutaraldehyde, and then, the cells were subsequently dehydrated using a graded ethanol series (30%, 50%, 70%, 80%, 90%, and 100%). After lyophilization and vacuum sputter‐coating with approximately 5 nm of gold/palladium (Macklin, Shanghai, China), the samples were observed in a Hitachi SU8000 scanning electron microscope (Hitachi, Beijing, China) [25].

### Confocal laser scanning microscopy (CLSM)

The changes in biofilm formation of USA300 after treatment with baicalin and oxacillin sodium, either individually or in combination, were observed using confocal laser scanning microscopy. Log phase bacteria were diluted to 2 × 10^7^ CFU·mL^−1^ and incubated in confocal culture dishes (bottom diameter 20 mm) at 37 °C for 18 h with various treatments. These treatments included sterile water (negative control), oxacillin sodium (1/4 × MIC and 1 × MIC), baicalin (1/4 × MIC), and combinations of oxacillin sodium (1/4 × MIC and 1 × MIC) with baicalin (1/4 × MIC). After three washes, the dead and live cells were assessed using the LIVE/DEAD BacLight™ bacterial viability kit (Invitrogen, Life Technologies, Shanghai, China) and observed under a confocal laser scanning microscope (CLSM 710, Carl Zeiss, Jena, Thuringia, Germany) [37]. The excitation/emission wavelengths for SYTO9 and propidium iodide (PI) were 480/500 and 490/635 nm, respectively. SYTO9 was used to stain live bacteria, while PI was used to stain dead bacteria.

### Biofilm assay

The effect of combined treatment on biofilm formation of USA300 was assessed using the microdilution method [38]. Briefly, 100 μL of the bacterial solution (2 × 10^5^ CFU·mL^−1^) was placed in a 96‐well microtiter plate with the tested agents (sterile water; 1/4 × MIC baicalin alone; 1/4 × MIC, 1 × MIC, 2 × MIC oxacillin sodium alone; 1/4 × MIC baicalin was combined with 1/4 × MIC, 1 × MIC, 2 × MIC oxacillin sodium, respectively), and the plate was incubated at 37 °C for 24 h. Wells containing biofilms were washed three times, fixed with methanol for 15 min, and stained with 0.1% (w/v) crystal violet (CV) for 5 min. After thorough washing, 95% ethanol was added to each well, and the plate was gently agitated at room temperature for 30 min. Gentamicin was used as a positive control. Biofilm biomass was measured at 590 nm using a microtiter plate reader (Spark™ multimode microplate reader, Tecan (Shanghai) Laboratory Equipment Co.). Biofilm biomass was calculated using the formula: OD590 of the sample/OD590 of the untreated control [8].

The 1‐day‐old biofilm was prepared by the method of Li *et al*. [39]. After 24 h of biofilm growth, the biofilms were washed with phosphate‐buffered saline (PBS) and then treated with the test agents (sterile water; 1/4 × MIC baicalin alone; 1/4 × MIC, 1 × MIC, 2 × MIC oxacillin sodium alone; 1/4 × MIC baicalin was combined with 1/4 × MIC, 1 × MIC, 2 × MIC oxacillin sodium, respectively), incubating at 37 °C for another 24 h. Subsequently, the biofilms were fixed, stained, and quantified as described above [38]. Gentamicin was used as a positive control.

### Congo red broth method

The content of polysaccharide intercellular adhesin (PIA) in USA300 biofilm was determined by Congo red (CR) method [40]. Briefly, bacterial cultures were diluted to 2 × 10^7^ CFU·mL^−1^, added with CR with a final concentration of 1 mg·mL^−1^, and incubated with different drug groups (sterile water; 1/4 × MIC baicalin alone; 1/4 × MIC, 1 × MIC, 2 × MIC oxacillin sodium alone; 1/4 × MIC baicalin was combined with 1/4 × MIC, 1 × MIC, 2 × MIC oxacillin sodium, respectively) at 37 °C for 48 h. Gentamicin was used as a positive control. CR binds to cellulose produced by the bacteria, forming a black‐red precipitate. The amount of precipitate indirectly reflects the PIA content.

### Statistical analysis

All experiments were repeated at least three times. The results have been expressed as mean ± standard deviation (SD). A paired Student's *t*‐test was used to test for significance. Significance is indicated as fellows: * for *P <* 0.05, ** for *P <* 0.01, *** for *P* < 0.001.

## Results

### MICs of baicalin and oxacillin sodium for USA300

The MIC of baicalin for USA300 was 1250 μg·mL^−1^. The minimum inhibitory concentration (MIC) values for USA300 against oxacillin sodium and gentamicin are 31.3 and 1.56 μg·mL^−1^, respectively. According to the National Committee for Clinical Laboratory Standards (NCCLS) standards for drug susceptibility testing [41], USA300 showed significant resistance to oxacillin sodium, while remaining susceptible to gentamicin.

### Synergistic antibacterial activity of baicalin and oxacillin sodium

This study explored the synergistic antibacterial effect between plant extracts and antibiotics by combining baicalin with oxacillin sodium, gentamicin. As shown in Table 1, baicalin was able to reduce the MIC of oxacillin sodium against MRSA USA300 strains by fourfold. Compared with oxacillin sodium alone, higher antibacterial activity with the lower MIC was observed when oxacillin sodium was cotreated with baicalin, which indicated that the presence of baicalin enhanced the effectiveness of oxacillin sodium. Gentamicin, used as a positive control, also demonstrated synergistic antimicrobial activity when combined with baicalin.

**Table 1:** Minimum inhibitory concentration (MIC) and fractional inhibitory concentration index (FICI) of baicalin, oxacillin sodium, and gentamicin against MRSA USA300. Values are means of three independent experiments. Index interpretation: ≤ 0.5, synergy; > 0.5 to 1, additive effect; > 1 to < 4, no effect; ≥ 4, antagonism.

Strains	Agents	MIC (μg·mL^−1^)		FICI	Outcome	Agents	MIC (μg·mL^−1^)		FICI	Outcome
		Alone	Combination				Alone	Combination		
USA300 (intact bacteria)	Baicalin	1250	312.5	0.5	Synergy	Baicalin	1250	39	0.2812	Synergy
	Oxacillin sodium	31.3	7.825			Gentamicin	1.56	0.39		
USA300 (pro toplasts)	Baicalin	312.5	62.5	0.325	Synergy	Baicalin	312.5	1.2	0.0191	Synergy
	Oxacillin sodium	1.956	0.245			Gentamicin	0.195	0.003		

### Growth curves and bactericidal kinetics of USA300 treated with combined baicalin and oxacillin sodium

**Fig. 1:**
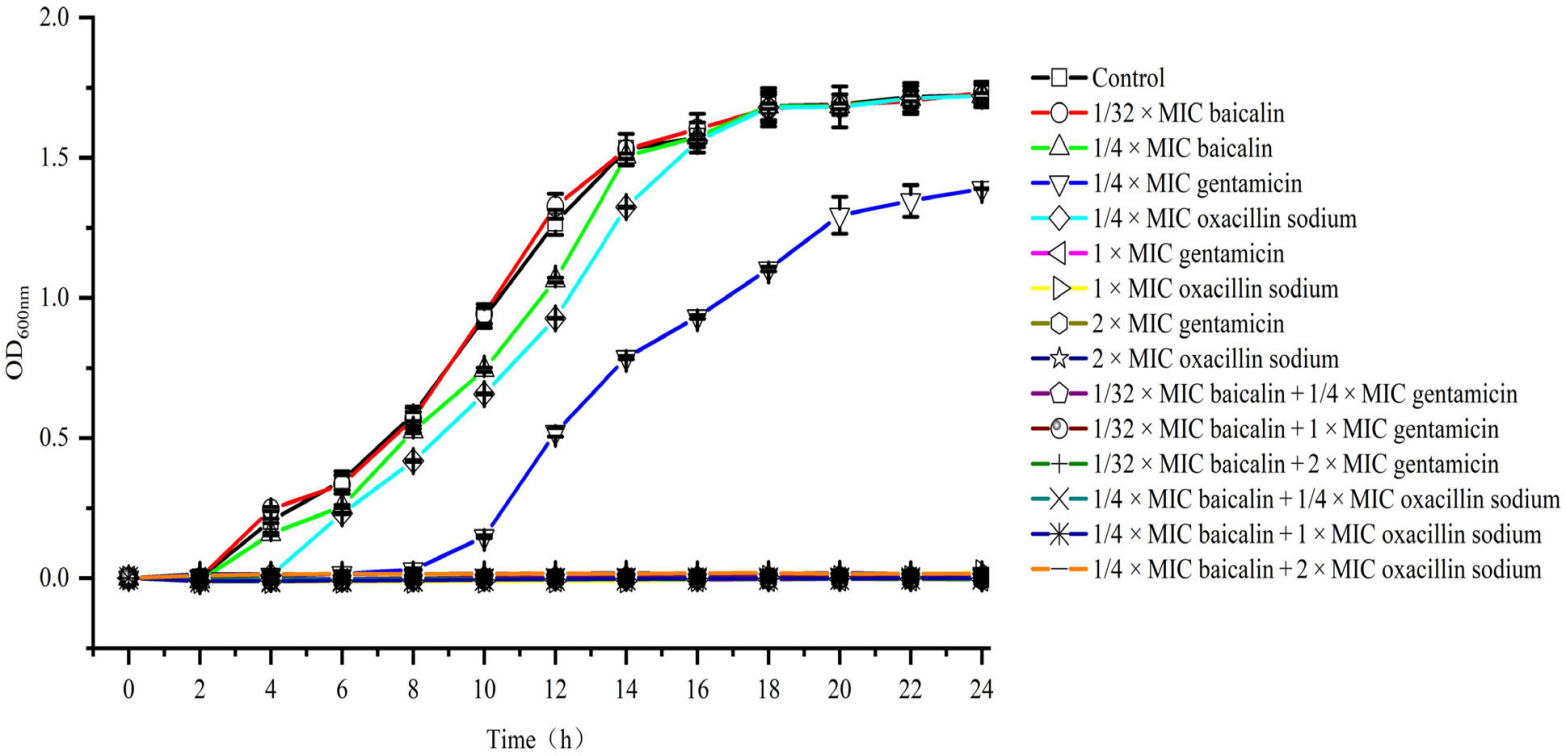
Growth curves of bacteria treated with baicalin and oxacillin sodium. Error bars represent mean ± SD of at least three independent experiments.

The growth curve results indicated that when 312.5 μg·mL^−1^ (1/4 × MIC) of baicalin or 1/4 × MIC of oxacillin sodium was used alone, bacterial growth was observed after 2 and 4 h, respectively. However, when 312.5 μg·mL^−1^ of baicalin was combined with 1/4 × MIC or higher concentrations (1 × MIC and 2 × MIC) of oxacillin sodium, bacterial growth was completely inhibited within 24 h (Fig. 1). Baicalin and gentamicin show similar antibacterial activity against methicillin‐resistant *Staphylococcus aureus* (MRSA) USA300 when used in combination. When treated with gentamicin at 1/4 × MIC alone, the bacteria began to show a growth trend after 10 h. However, when baicalin at 39 μg·mL^−1^ (1/32 MIC) was used in combination with varying concentrations (1/4 × MIC, 1 × MIC, and 2 × MIC) of gentamicin, bacterial growth was completely inhibited (Fig. 1).

### Bactericidal kinetics of USA300 treated with combined baicalin and oxacillin sodium

**Fig. 2:**
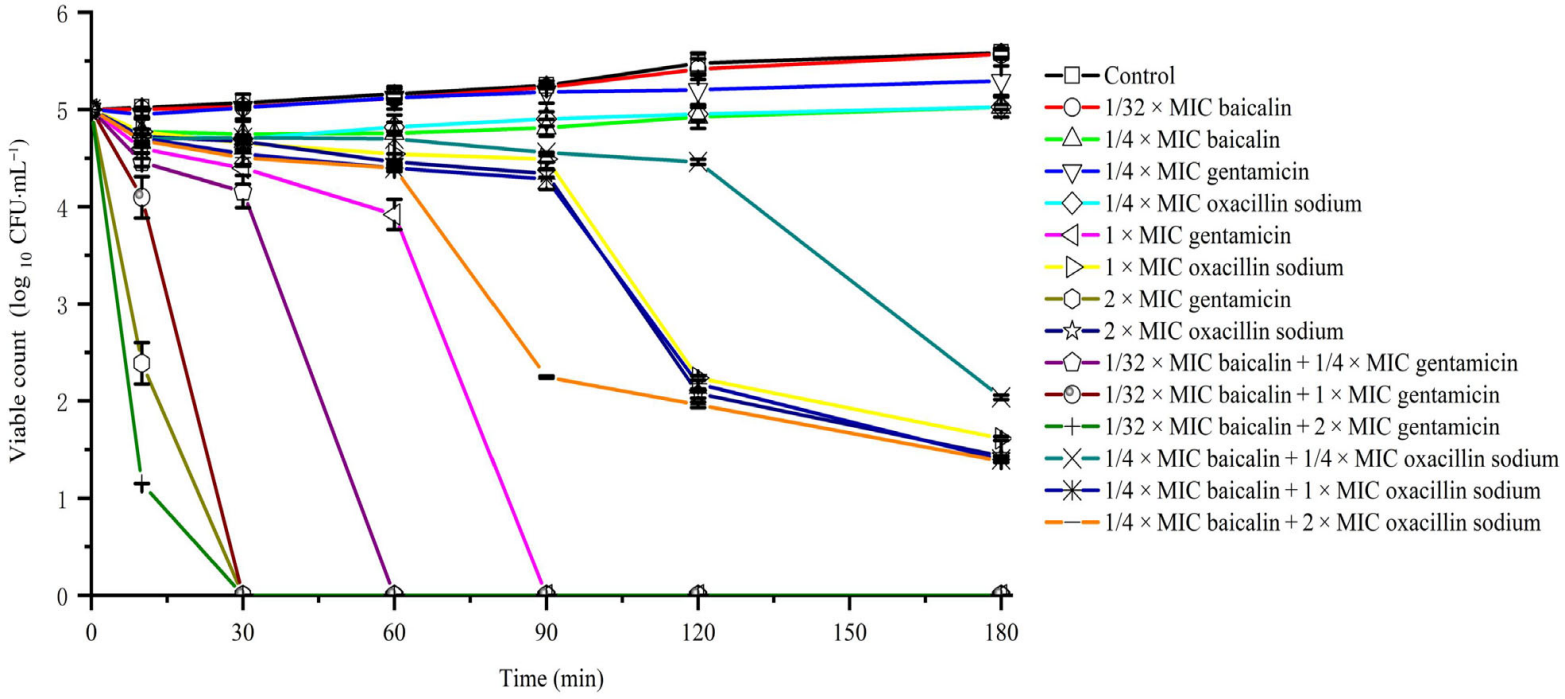
Time–kill curves of bacteria treated with baicalin and oxacillin sodium. Error bars represent mean ± SD of at least three independent experiments.

The bactericidal kinetics results demonstrated that the synergistic bactericidal activity of baicalin and oxacillin sodium was both dose‐ and time‐dependent. As shown in Fig. 2, the number of untreated bacteria increased for 180 min. Oxacillin sodium at 1/4 × MIC alone was ineffective in inhibiting bacterial growth, with bacteria showing growth after just 10 min. However, when combined with 312.5 μg·mL^−1^ (1/4 × MIC) baicalin, bacterial growth was inhibited, reaching maximum inhibition at 180 min, with a reduction of more than 3 log_10_ CFU·mL^−1^. Increasing the concentration of oxacillin sodium enhanced the bactericidal effect. Oxacillin sodium at 2 × MIC alone reduced bacterial counts by 3 log_10_ CFU·mL^−1^ in 120 min, whereas the combination of 2 × MIC oxacillin sodium and 1/4 × MIC baicalin achieved the same reduction in only 90 min. These findings indicated that baicalin enhanced the bactericidal rate and efficacy of oxacillin sodium. In addition, the concurrent use of gentamicin and baicalin demonstrates a more potent bactericidal effect. Compared to 1/4 × MIC gentamicin alone, the combination with 39 μg·mL^−1^ baicalin completely eradicates USA300 within 60 min.

### Effects of baicalin combined with oxacillin sodium on the cell membrane and cell wall permeability of USA300

**Fig. 3:**
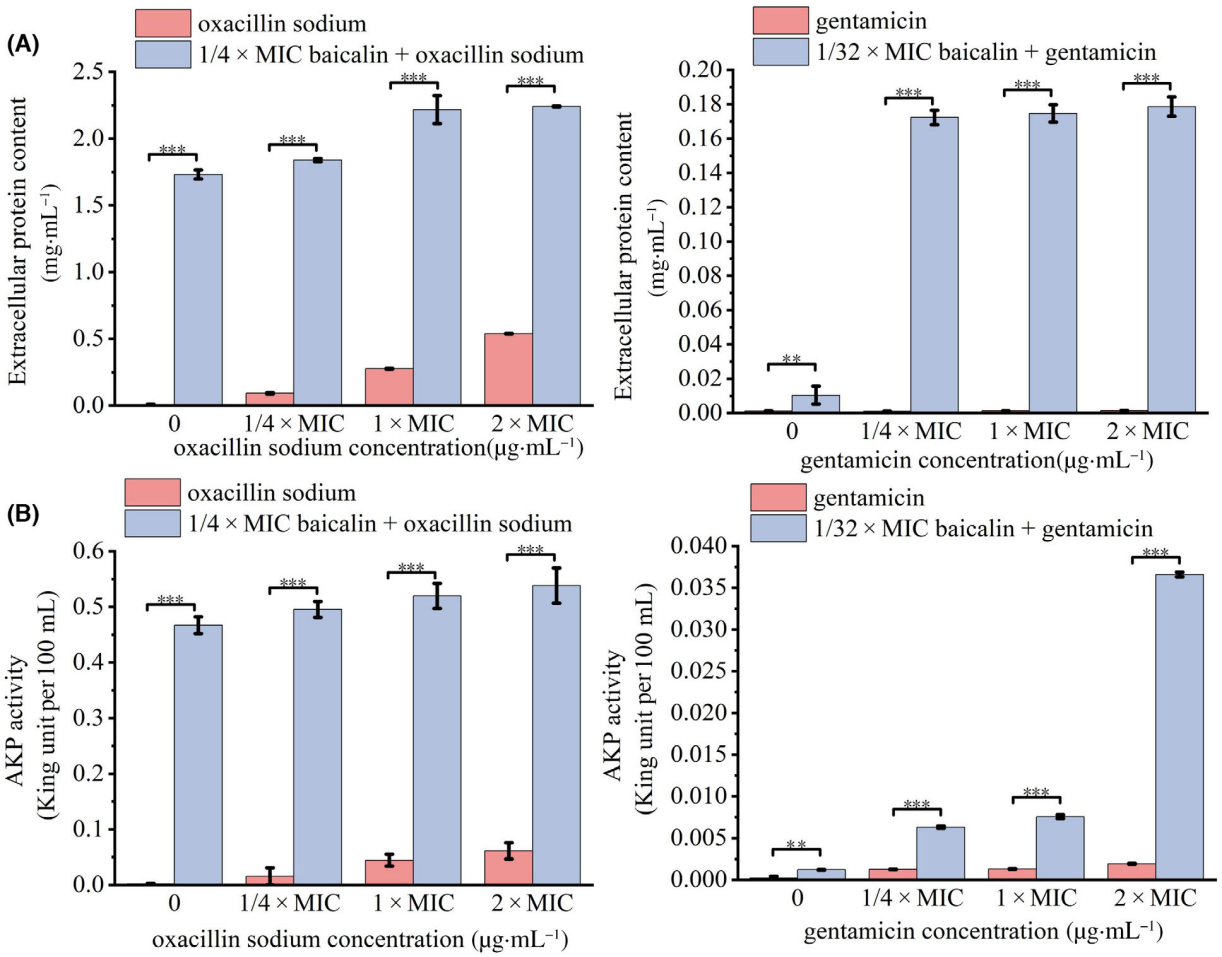
Effects of baicalin combined with oxacillin sodium on USA300 cell membrane and cell wall permeability. (A) Extracellular protein content of USA300 cells in the presence of baicalin and oxacillin sodium was detected by BCA protein detection kit, as described in the Materials and methods section. (B) Changes in cell wall permeability of USA300 cells induced by baicalin and oxacillin sodium. AKP is a cytoplasmic enzyme released only from cells with compromised cell wall permeability, and extracellular AKP activity was measured using AKP viability assay kit. Error bars represent mean ± SD of at least three independent experiments. Statistical analysis was conducted using the *t*‐test method. ** represents *P* ≤ 0.01 and *** represents *P* ≤ 0.001.

The changes in bacterial cell membrane permeability due to the combined treatment of baicalin and oxacillin sodium were evaluated by the release of extracellular protein content. The results indicated that the combined treatment significantly increased protein release, compared to oxacillin sodium alone and the control group (0.01 mg·mL^−1^) (Fig. 3A). Treatment with 1/4 × MIC baicalin, 1/4 × MIC oxacillin sodium, and their combination resulted in protein leakage levels of 1.73, 0.09, and 1.84 mg·mL^−1^, respectively. When the concentration of oxacillin sodium was increased to 1 × MIC or 2 × MIC and combined with baicalin, the leakage of intracellular bacterial proteins increased from 0.28 to 2.22 mg·mL^−1^ (compared with 1 × MIC oxacillin sodium alone) and from 0.54 to 2.24 mg·mL^−1^ (compared with 1 × MIC oxacillin sodium alone), respectively. Additionally, baicalin alone induced significantly more bacterial cell protein leakage than oxacillin sodium alone, with 1/4 × MIC baicalin causing protein leakage 3.2 times greater than that caused by 2 × MIC oxacillin sodium (Fig. 3A). Additionally, the combination of baicalin and gentamicin exhibits a stronger effect on the leakage of intracellular proteins from USA300 cells. The results showed that baicalin played a primary role in disrupting the bacterial cell membrane and increasing membrane permeability during their combined treatment.

AKP is typically released only from cells with impaired cell wall permeability, so measuring AKP activity can indirectly assess the impact of baicalin and oxacillin sodium on bacterial cell wall permeability [42]. Figure 3B showed that the extracellular AKP activity of untreated USA300 cells was 0.002 King μnits per 100 mL. Compared to the control group, baicalin alone, oxacillin sodium alone, gentamicin alone, and the combinations of baicalin with oxacillin sodium or gentamicin all led to an increase in extracellular AKP levels. Notably, the combination of baicalin and oxacillin sodium was more effective in disrupting the cell wall and promoting AKP release than oxacillin sodium alone. The extracellular AKP activity measured was 0.5 King μnits per 100 mL when cells were treated with the combination of 1/4 × MIC oxacillin sodium and 1/4 × MIC baicalin, which was 250 times higher than the untreated group and 25 times higher than with 1/4 × MIC oxacillin sodium alone. Thus, baicalin enhanced the permeability effect of oxacillin sodium on the cell wall, promoting AKP release.

To further investigate the role of the cell wall in the combined antimicrobial activity of baicalin and oxacillin sodium against USA300, we prepared protoplasts. Compared to intact USA300 cells, protoplasts lacking a cell wall showed higher sensitivity to all treatments. Specifically, the MIC of baicalin, oxacillin sodium, and gentamicin alone for USA300 protoplasts decreased by 4‐fold (from 1250 μg·mL^−1^ down to 312.5 μg·mL^−1^), 16‐fold (from 31.3 μg·mL^−1^ down to 1.956 μg·mL^−1^), and 8‐fold (from 1.56 μg·mL^−1^ down to 0.195 μg·mL^−1^) respectively. When oxacillin sodium or gentamicin is used in combination with baicalin, compared to intact USA300 cells, the MIC of oxacillin sodium and gentamicin for USA300 protoplasts is reduced by 32‐fold (from 7.825 μg·mL^−1^ down to 0.245 μg·mL^−1^) and 130‐fold (from 0.39 μg·mL^−1^ down to 0.003 μg·mL^−1^), respectively (Table 1). Thus, the increased sensitivity of USA300 protoplasts to the combination of baicalin and oxacillin sodium or gentamicin highlighted the significant role of the cell wall in the antimicrobial activity of this combination.

### SEM analysis

**Fig. 4:**
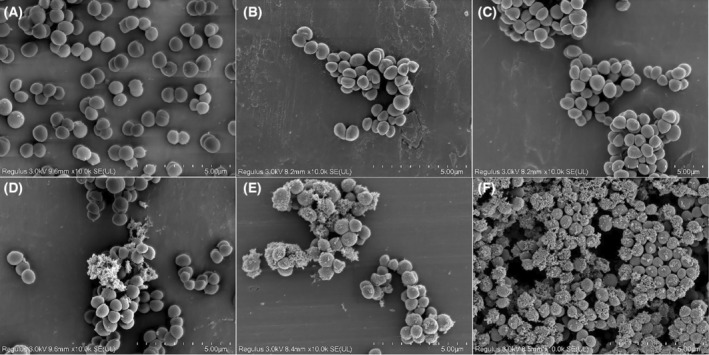
Scanning electron micrograph of USA300 cells. USA300 cells were treated with TSB medium (A), 1/4 × MIC of baicalin (B), 1/4 × MIC (C) and 1 × MIC (E) of oxacillin sodium, the combination of 1/4 × MIC of baicalin and 1/4 × MIC of oxacillin sodium (D) or 1/4 × MIC of baicalin and 1 × MIC of oxacillin sodium (F) at 37 °C for 1 h. After washing, the bacterial cultures were fixed, dehydrated and coated with gold/palladium, and then observed under a scanning electron microscope, as described in the Materials and methods section. The images are representative of at least three independent experiments.

Representative SEM images of the USA300 strain treated with oxacillin sodium, baicalin, or both confirmed the disruption of bacterial membranes (Fig. 4). Untreated USA300 cells showed intact morphology, smooth surfaces, and clear edges. When treated with 1/4 × MIC of oxacillin sodium or 1/4 × MIC of baicalin alone, the bacterial morphology showed no changes compared to the control group, with clear edges and no leakage of contents. However, the combination of 1/4 × MIC oxacillin sodium and 1/4 × MIC baicalin caused the bacterial cell surface to rupture and release cell contents. Furthermore, when 1/4 × MIC baicalin was combined with 1 × MIC oxacillin sodium, it caused more pronounced shrinkage, rupture, and leakage of bacterial cell contents compared to the 1 × MIC oxacillin sodium alone. These findings demonstrated that the synergistic effect of oxacillin sodium and baicalin enhanced the destruction of the bacterial membrane, leading to cell death.

### Antibiofilm activity of baicalin combined with oxacillin sodium

**Fig. 5:**
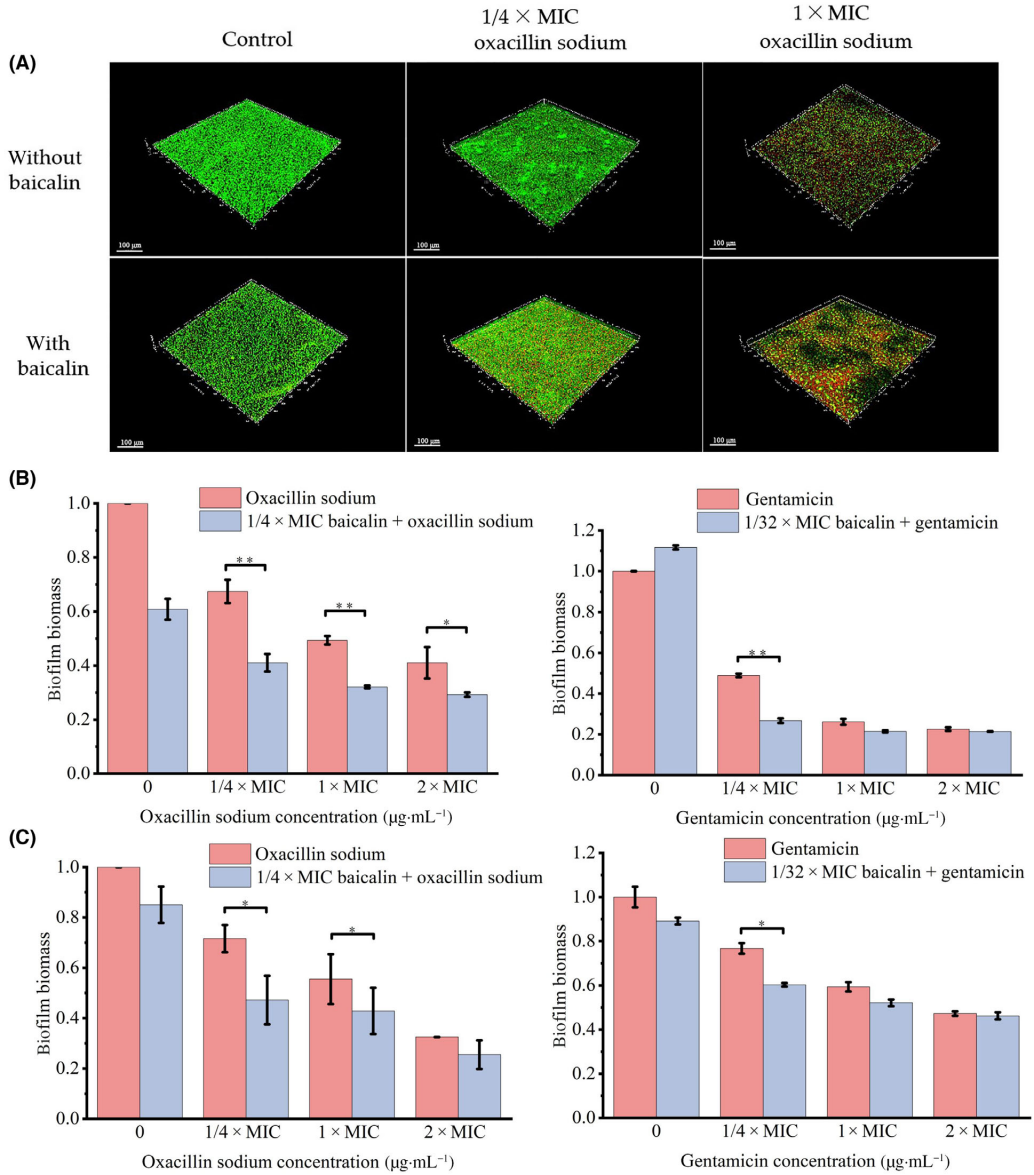
Antibiofilm activity of combination of baicalin and oxacillin sodium. (A) Three‐dimensional reconstructions of USA300 biofilms were created using CLSM. The combination of baicalin and oxacillin sodium inhibits biofilm formation (B) and decomposition of 1‐day‐old biofilm (C). Error bars represent mean ± SD of at least three independent experiments. Statistical analysis was conducted using the *t*‐test method (*P < 0.05 and **P < 0.01).

USA300 biofilms were incubated for 24 h with baicalin, oxacillin sodium, or their combination, and images were recorded using CLSM after staining. As shown in Fig. 5A, 1/4 × MIC baicalin had no bactericidal effect on USA300, resulting in green fluorescence similar to the control group. Oxacillin sodium (1/4 × MIC) also did not penetrate the biofilm effectively, predominantly showing green fluorescence. The combination of baicalin and oxacillin sodium with different concentrations enhanced the red fluorescence (representing dead cells). Notably, the combination of 1 × MIC oxacillin sodium with baicalin (1/4 × MIC) exhibited stronger red fluorescence, indicating that the combination can significantly reduce the biomass of USA300 biofilm.

To further confirm the antibiofilm effect of the combination of baicalin and oxacillin sodium, quantitative analysis was carried out by crystal violet staining. As shown in Fig. 5B, 1/4 × MIC oxacillin sodium alone resulted in a 33% inhibition rate of USA300 biofilm formation. When combined with baicalin, 1/4 × MIC oxacillin sodium significantly enhanced the inhibition of biofilm formation, reaching a 58% inhibition rate. The efficacy of the combination improved with increasing oxacillin sodium concentrations. 2 × MIC oxacillin sodium alone achieved a 59% inhibition rate, which increased by 12% when combined with 1/4 × MIC baicalin. Moreover, the combination of baicalin with different concentrations of oxacillin sodium also enhanced degradation of 1‐day‐old USA300 biofilms. Compared to oxacillin sodium alone, combining various concentrations of oxacillin sodium with baicalin increased the biofilm degradation rate of 1‐day‐old biofilms by 7–24% (Fig. 5C). Similarly, the combination of baicalin and gentamicin also promotes the inhibition and degradation of USA300 biofilm (Fig. 5B,C). These results indicated that baicalin effectively enhanced the inhibitory effect of antibiotics on both the formation and degradation of USA300 biofilms.

### Evaluating the effects of baicalin combined with oxacillin sodium on PIA in USA300 biofilms

**Fig. 6:**
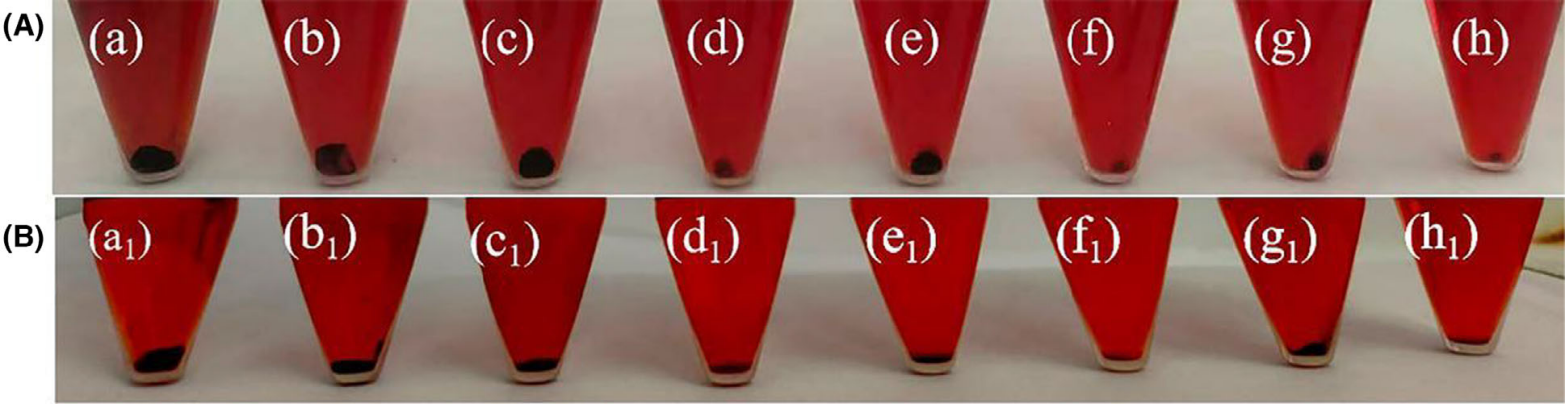
Effect of combination of baicalin and oxacillin sodium (A) or gentamicin (B) on the synthesis of biofilm component PIA. USA300 cell were grown for 48 h in TSB liquid medium containing CR as control (a and a1) or in the presence of baicalin alone (b and b1), different concentrations of oxacillin sodium alone (c, e, g) or in combination with baicalin (d, f, h), different concentrations of gentamicin alone (c1, e1, g1), or in combination with baicalin (d1, f1, h1). Among them, (b): 1/4 × MIC baicalin; (c): 1/4 × MIC oxacillin sodium; (d): 1/4 × MIC baicalin + 1/4 × MIC oxacillin sodium; (e): 1 × MIC oxacillin sodium; (f): 1/4 × MIC baicalin + 1 × MIC oxacillin sodium; (g): 2 × MIC oxacillin sodium; (h): 1/4 × MIC baicalin + 2 × MIC oxacillin sodium; (b1):1/32 × MIC baicalin; (c1): 1/4 × MIC gentamicin; (d1): 1/32 × MIC baicalin + 1/4 × MIC gentamicin; (e1): 1 × MIC gentamicin; (f1): 1/32 × MIC baicalin + 1 × MIC gentamicin; (g1): 2 × MIC gentamicin; (h1): 1/32 × MIC baicalin + 2 × MIC gentamicin. PIA was assessed using the Congo red method, as described in the Materials and methods section. The results of PIA were repeated three times.

PIA is the primary extracellular polysaccharide component of *Staphylococcus aureus* biofilms and plays a crucial role in biofilm formation [43]. Congo red has a particular affinity for β‐1,3 and β‐1,4 glucans, which are structural components of many bacterial extracellular polysaccharides [44]. Congo red binds to PIA when it is produced, resulting in a black‐red precipitate. As shown in Fig. 6, the control group produced the most black‐red precipitate. Compared to oxacillin sodium alone, the combination of baicalin with oxacillin sodium resulted in less black‐red precipitate. The combined use of gentamicin and baicalin also shows similar effects. This indicated that the combination more effectively inhibited the formation of the biofilm component PIA, thereby destabilizing the biofilm structure and promoting bacterial cell death.

## Discussion

Traditional Chinese medicine extracts are less likely to induce resistance compared to current antibiotics due to their antibacterial activity and unique mechanisms targeting bacterial membranes [45, 46, 47, 48]. Studies have shown that combining herbal extracts with conventional antibiotics can synergistically enhance antimicrobial effects on MRSA, lower the minimum inhibitory concentration (MIC) of antibiotics, and delay the development of resistance [49, 50, 51]. The present study confirmed the synergistic effect of baicalin and oxacillin sodium against the USA300 strain (Table 1). Oxacillin sodium alone exhibited weak antibacterial activity against USA300, with a MIC of 31.3 μg·mL^−1^. However, when combined with 1/4 × MIC of baicalin, the antibacterial activity of oxacillin sodium significantly increased, reducing the MIC by 4‐folds (Fig. 1). The bacterial growth curve results further illustrated that oxacillin sodium at 1/4 × MIC completely inhibited bacterial growth when used in synergy with baicalin at 1/4 × MIC, which could not be achieved by oxacillin sodium alone (Fig. 2A). Moreover, compared to oxacillin sodium alone, the combination treatment with baicalin significantly accelerated the bactericidal rate and enhanced antibacterial efficacy (Fig. 2B).

Most traditional Chinese medicine extracts cause bacterial death by disrupting the cell membrane or cell wall [52, 53, 54, 55]. In this study, we investigated whether the combination of baicalin and oxacillin sodium resulted in the disruption of bacterial cell walls and cell membranes. Protein leakage assays revealed that the combination of baicalin and varying concentrations of oxacillin sodium caused intracellular bacterial protein leakage 4 to 20 times higher than oxacillin sodium alone, indicating a synergistic effect in promoting protein leakage (Fig. 3A). Alkaline phosphatase (AKP) activity is commonly used as an important indicator of cell wall integrity [56]. An increase in AKP activity in the extracellular environment suggests that the cell wall has ruptured, resulting in the leakage of AKP and other intracellular contents [56]. Our results showed that the activity of AKP after treatment with 1/4 × MIC oxacillin sodium alone did not significantly differ from the control group, indicating that low concentrations of oxacillin sodium do not affect bacterial cell wall permeability (Fig. 3B). However, when combined with 1/4 × MIC baicalin, there was a significant increase in the permeability of the USA300 cell wall (Fig. 3B). In addition, when the cell wall was removed from USA300 cells, the resulting protoplasts exhibited high sensitivity to oxacillin sodium. Compared to intact USA300 cells, the MIC values of oxacillin sodium alone and in combination with baicalin for USA300 protoplasts decreased by 16‐ and 128‐fold (Fig. 3C), respectively. SEM images further confirmed that the combined treatment of baicalin and oxacillin sodium disrupted the bacterial membrane of the USA300 strain (Fig. 4). These results suggested that the synergistic action of baicalin and oxacillin sodium may be attributed to baicalin mediating the disruption of cell membrane and cell wall integrity, enhancing the uptake of oxacillin sodium by bacterial cells, thereby achieving higher local drug concentrations to effectively eradicate bacteria. The above results are basically consistent with the statement proposed by Liu *et al*. [57], that the synergistic mechanism between TCM extracts and antibiotics lies in the destruction of bacterial membranes mediated by TCM extracts, which helps antibiotics to enter cells more easily, thus reducing the resistance of drug‐resistant bacteria to antibiotics.

Bacterial biofilm is a protective film formed on the surface of attached carrier by macromolecular substances such as polysaccharide intercellular adhesion (PIA) secreted by bacteria [58, 59]. Compared to planktonic cells, bacteria within biofilms exhibit greater resistance to antibiotics [60]. We found that the combination of baicalin and oxacillin sodium more effectively reduced the biomass of existing USA300 biofilms compared to oxacillin sodium alone by enhancing the inhibition and degradation of biofilm formation (Fig. 5). Yang *et al*. [61] also reported a synergistic effect in combating bacterial biofilms when combining traditional Chinese medicine extracts with antibiotics. PIA is the main component of *Staphylococcus aureus* biofilm, which can mediate interbacterial adhesion and maintain the stability of biofilm structure [62]. Our study found that the combination of baicalin and oxacillin sodium effectively reduced the formation of PIA, providing direct evidence that baicalin combined with oxacillin sodium can mediate the diffusion of the biofilm matrix (Fig. 6).

## Conclusion

Our study reveals the synergistic activity of baicalin combined with oxacillin sodium against USA300 *in vitro*. This combination therapy maintains antimicrobial activity while reducing the dosage of conventional antibiotics, helping to delay the emergence of resistance. Bacterial cell membrane and cell wall are the main targets of baicalin. Baicalin can lead to increased cell membrane permeability and loss of barrier function, allowing traditional antibiotics to penetrate cells easily and effectively exert antibacterial activity. Therefore, our research indicates that the combined use of baicalin with oxacillin sodium antibiotic helps to reduce MRSA's antibiotic resistance. Future research plans will delve into the mechanism of action of baicalin and oxacillin sodium in combination against MRSA biofilms based on the quorum sensing system.

## Conflict of interest

The authors declare no conflict of interest.

## Author contributions

XM and HG conceived the study, designed experiments, interpreted the data, and wrote the manuscript. MK, ZY, and YC performed the majority of the experiments and analyzed the results. XM supervised the study and provided new tools and reagents. CL performed the experiments and statistical analysis. TJ and KW provided expertise for data interpretation.

## Supporting information


**Fig. S1.** Standard curve of MRSA USA300 inoculum corresponding to OD600nm measurement values.

## Data Availability

The data that support the findings of this study are available from the first author upon reasonable request.
