# Supplementary material for: Synergistic antibacterial activity of baicalin in combination with oxacillin sodium against methicillin‐resistant Staphylococcus aureus
Source: FEBS Open Bio. 2024 Dec 15;15(4):608–21. doi: 10.1002/2211-5463.13952 (PMC11961378; doi:10.1002/2211-5463.13952)
Supplement: Supplementary file 1 — Fig. S1. Standard curve of MRSA USA300 inoculum corresponding to OD600nm measurement values. [file FEB4-15-608-s001.pdf]

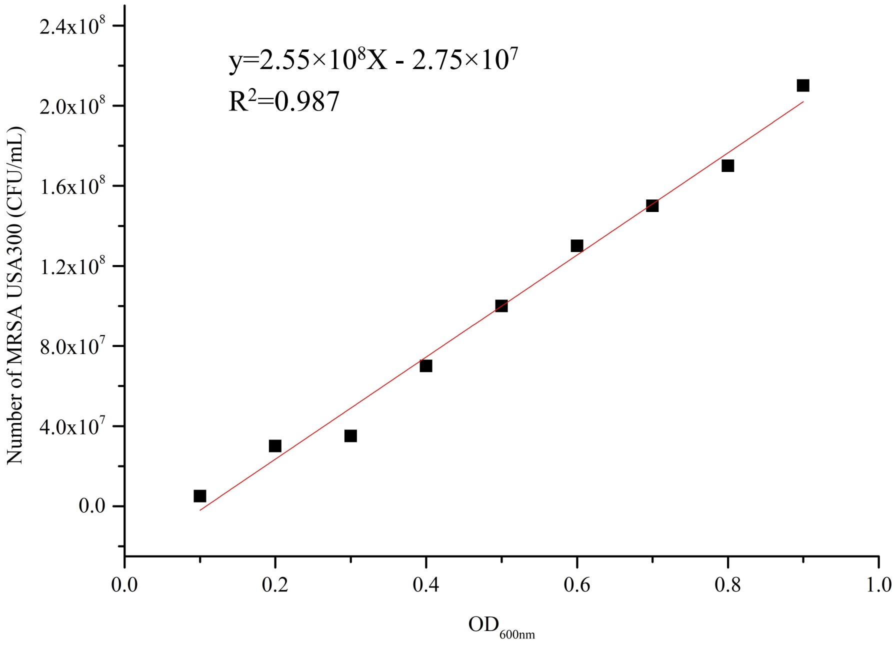

Fig. S1. Standard curve of MRSA USA300 inoculum corresponding to OD600nm measurement values [1].

References

[1] Lucidi M, Marsan M, Pudda F, Pirolo M, Frangipani E, Visca P, Cincotti G (2019). Geometrical-optics approach to measure the optical density of bacterial cultures using a LED-based photometer. Biomed Opt Expres 10, 5600-5610.
